# Kept at a Distance: A Qualitative Study of Bereaved Individuals’ Experiences of How Death Was Addressed When Their Partner Died at Home

**DOI:** 10.1177/10497323251328294

**Published:** 2025-03-27

**Authors:** Margareta Aurén-Møkleby, Gunvor Aasbø, Anne Marit Mengshoel, Kari Nyheim Solbrække, Lisbeth Thoresen

**Affiliations:** 1 Institute of Health and Society, 6305 University of Oslo, Oslo, Norway

**Keywords:** awareness of dying, end-of-life communication, couples, death and dying, end-of-life issues, death at home, qualitative research

## Abstract

Open awareness and dialogue concerning dying are considered essential for planning and realizing death at home. Moreover, much help and support throughout the dying process and death trajectory are provided by a person’s next of kin, often a spouse or partner. To explore how death was addressed among couples when one of the partners had died at home, we interviewed 14 bereaved individuals. The results were grouped into the following themes: “Idea(l)s and realities of communication,” “Different kinds of talks,” and “Unspoken understandings and showing without talking.” We found that prevailing narratives about how death should be discussed in socially and culturally expected ways affected how the bereaved addressed the imminent death of their partner, both at the time and in their retrospective reflections. In a few cases, death had been talked about directly using words such as “death” and “dying,” although indirect or avoidant discussions about death were more common. The bereaved mentioned unspoken understandings about how it was unnecessary to address death—that is, one just knew. In cases where the couple could not share a silent understanding, the bereaved had experienced loneliness. Death could also be addressed through actions, such as becoming closer or the ill person arranging for an easier subsequent life for their partner. To reduce the expectations that dying people and their partners might have to interact in certain ways at the end of life, it is important to acknowledge that awareness of dying can be expressed and shared in various ways.

## Introduction

“Do you guys ever think about dying?” When Barbie utters this question during a dance party, the music suddenly stops and everybody freezes (Gerwig, 2023). A fun and relaxed gathering becomes awkward, and that feeling lasts long enough to cause uneasiness, before Barbie saves the situation by saying “I don’t know why I just said that … I’m just … dying to dance!” The music restarts and the dance, and life, goes on, although the expression on Barbie’s face suggests that her mind is partly elsewhere. The time and place she chose to discuss death were perhaps unusual, but Barbie did what is often encouraged—that is, she acknowledged and was open about death being a natural part of life.

Talking about death and dying can prove difficult for most of us, even in situations where severe illness means a limited lifespan and an imminent experience of death is a reality for those involved. In previous studies that explored death at home from the perspectives of dying patients, their life partners, and bereaved individuals, we found that death and dying were rarely spoken of between those involved (Aurén-Møkleby et al., 2023, 2024). The data material hence challenged a Western contemporary idea(l) emphasizing awareness and openness concerning death (Clark & Seymour, 1999; Seale et al., 1997), especially when life is ending and home is the preferred place to die. This article focuses on exploring how death and dying were addressed within couples when one of the partners died at home from cancer.

### Background

The way in which death has been perceived and organized has changed throughout history. Indeed, ideals and ways of handling death can be understood as mirroring the mentalities and practices of different periods (Ariès, 1976a; Jacobsen, 2020; Walter, 2017). Typologies, such as modern and post-modern forms of death (Walter, 1994) and Philippe Ariès’ (1976b) historical phases of dying and death in the context of the Western world from the Middle Ages to the 20th century, should be understood as simplified notions of how death has been conceptualized and managed throughout history (Clark & Seymour, 1999). Ariès has been criticized for his “selection of facts” (Elias, 1985, as cited in Clark & Seymour, 1999) and for presenting a somewhat romanticized view of dying as familiar and “normal” in earlier times (Clark & Seymour, 1999). After World War II, there was a significant demographic and epidemiological shift marked by population growth, increased life expectancy, and a transition from infectious diseases to cancer as the leading cause of death. From the 1950s onward, changing attitudes toward death paralleled advancements in modern medicine and hospital care (Clark & Seymour, 1999), the emergence of psychological theories (Seale, 1998), and the rise of secularization and values emphasizing individuality and autonomy (Taylor, 1992). The place for death changed from what Ariès describes as “at home in the bosom of one’s family” (1976a, p. 87) to dying at a hospital in solitude. Through moving the dying person to the hospital, death and dying were rendered hidden and forbidden (Ariès, 1976a), while “a conspiracy of silence” surrounded the dying patient during the 1960s, especially if the patient was dying from cancer (Seale, 1998). Medical doctors at that time reported a preference for not informing a patient about their cancer diagnosis. This preference was related to cancer then connoting a certain death, a belief found among both medical doctors and patients. By informing a patient about a cancer diagnosis, a medical doctor risked depriving the patient of hope regarding recovery and survival (Novack, 1979).

Medical doctors’ monopoly on knowledge and information about serious illness, especially the trajectory of dying, is related to Glaser and Strauss’s (1965) concept of closed awareness. Their study highlighted the complexity of interactions and communications between dying patients, next of kin, and healthcare professionals (HCPs) in hospitals. Closed awareness indicated that the patient was not informed about their situation, whereas open awareness meant that all parties acknowledged and were open about the fact that the patient was dying. The respective mutual understanding set the scene for relations and interactions at the end of life. However, the authors acknowledged that “openness does not eliminate complexity” (Glaser & Strauss, 1965, p. 79).

In the 1960s and 70s, the hospice movement and death awareness movement arose in Western societies, promoting open awareness of death and how individuals and their next of kin should be involved in end-of-life care and engage with the death trajectory (Jacobsen & Dalgaard, 2013; Walter, 2017). The death awareness movement centered on an understanding of the dying individual as a person rather than “a useless and failed machine” (Kastenbaum, 1979, pp. 194–195). The idea(l)s of autonomy and self-realization became core values in end-of-life care and were strongly connected to the notion of a good death (Jacobsen & Dalgaard, 2013).

As medicine became less paternalistic, the requirements and expectations in the Western world regarding patients being active and making informed choices regarding their own health, illness, and treatment increased (Walter, 2017). The (cancer) patient was expected to be a responsible patient who knew what they wanted (Driller et al., 2022; Thoresen & Røberg, 2022). As part of this new patient role, open conversations concerning end-of-life planning when living with an incurable and life-threatening illness were encouraged (HospiceUK, 2024; Noonan et al., 2016; Sallnow et al., 2022). The patient-centered care perspective not only gave the patient the right to know their own condition, prognosis, and options but also made the patient “the gatekeeper of knowledge that may or may not be passed on to other family members” (Seale, 1998, p. 108).

As our study is conducted in Norway, it is particularly interesting to note how the idea(l) of openness concerning death is featured in Norwegian healthcare policies. An open dialogue about death is not only recommended by the authorities but specified to be “a prerequisite for understanding and accepting death as part of life”^1^ (Regjeringen, 2020, p. 8). The government’s view is built on the understanding that openness about dying is linked to planning during the end of life, admitting fear when facing imminent death, fostering emotional closeness, forgiving and resolving, and having the possibility of strengthening relationships with next of kin (Keeley, 2007; Seale et al., 1997). Studies on end-of-life interactions and conversations within families have revealed that family conversations are important but difficult due to possible disagreements and the physical progression of illness (Wallace, 2015), as well as how few people have experience of talking about death and how such conversations take place at an emotionally challenging time (Haaksman et al., 2024; Keeley, 2017). Furthermore, studies have shown how related interactions are multifaceted and complex, how families often strive for balance and well-being among all involved (Möllerberg et al., 2017), and how final conversations provide an opportunity for closeness, a possibility to say goodbye, and means of preparing the family for life after the death of their loved one (Häger Tibell et al., 2024; Keeley & Generous, 2017).

In Norway and other Western societies, there is a growing trend toward providing more healthcare services within the home, extending to end-of-life care (Ågren et al., 2023; Borgstrom & Walter, 2015; Regjeringen, 2020; Sallnow et al., 2022). End-of-life research has largely focused on individual experiences of patients and their caregivers, leaving a dearth of empirical studies exploring the experiences of couples during the end-of-life period (Gardner, 2008). To address a gap identified in the literature concerning how couples manage the experience of a partner dying at home, our study examines how discussions about death—or the lack thereof—take place within intimate partnerships or marriages when one partner passes away at home. Given the crucial role a spouse or partner plays in enabling the possibility of home death, understanding these dynamics is essential. Hence, the aim of this article is to explore how death was dealt with when one partner was seriously ill with cancer and died at home. Relatedly, we pose the following research question (RQ): How do bereaved individuals describe and reflect on the ways death was addressed when their ill partner spent the end of life at home and died there?

## Materials and Methods

### Study Design and Theoretical Perspective

This qualitative interview study drew on ideas from interactionism that emphasize how understandings and meanings are shaped within contexts and through social processes (Järvinen, 2017). Our starting point was that knowledge developed from interview data represents re- and co-constructions shaped by those involved, existing understandings, values, and time (Burr, 2015). Bereaved individuals who had experience of the death at home of a spouse or partner participated in individual open interviews. In the analytic process, we investigated how death and dying had been spoken of/not spoken of. Drawing on ideas from reflexive thematic analysis, we explored the nuances and complexities in each interview transcript, as well as across the data material, moving back and forth during the interpretation, engaging with diversities, contradictions, and possible uncertainties while seeing the larger picture and adopting a reflexive stance concerning our own positions (Braun & Clarke, 2022). During the generation of data, we were guided by the concept of information power addressing the dialogue quality in the interview setting, sample specificity, and analysis strategy (Malterud et al., 2016).

### Setting and Recruitment

We recruited participants in four ways—namely, through healthcare services, social media, personal networks, and snowball sampling (Braun & Clarke, 2013). The inclusion criteria were adults over the age of 18 who had experienced the death at home of a spouse or partner diagnosed with cancer. The inclusion criteria specified that participants must have experienced their partner’s death at home at least 6 months ago, but no more than 6 years ago. When HCPs were involved in the recruitment, they introduced the study to the bereaved individual after the death at home, and if the bereaved individual was interested, their contact information was passed to the first author. Otherwise, those who had read or heard about the study and wanted to participate contacted MAM or LT directly.

In total, 24 people expressed interest in the study. Two were excluded due to their relationship with the deceased and the length of time since the death at home occurred. Twenty-two people received written information about the study via e-mail. Eight did not respond or changed their mind about participating. Thus, 14 men and women participated in the study and were interviewed during the period February 2022 to August 2023.

For clarity’s sake, for the remainder of this article, the bereaved person will be referred to as the spouse, bereaved, or participant, while their ill, and now deceased, partner will be termed the partner. The results of this study are based on recollections and reflections by bereaved individuals, and what is presented is their perception and understanding of what happened.

### Participants

All the participants (*n* = 14) were of Norwegian ethnicity and aged between 49 and 81 years. Ten were women, and ten were retired. The participants’ present or former professions varied (healthcare workers, organizational and administrative workers, educational and cultural workers). They had been married or in a partnership with their now deceased partner for around 20–50 years. All had been in heterosexual relationships, and all had children. In most cases, the children were adults and lived on their own. In one case, the adult children had been temporarily living with their parents, while one family had children under the age of 18 years living at home at the time of the partner’s death.

Eight participants had lost their partner within the 8 months prior to the interview, four between one and a half to two years previously, and two had experienced the death at home of their partner 4 to 6 years ago. The varying lengths of time since participants experienced the death of their partner might have influenced the bereaved individuals’ experiences. However, this potential difference was not evident during the interviews, as all participants appeared to recall their experiences clearly. Some participants even referred to journals they had kept during that period, which helped them recount events during the interviews. The partners were 57–82 years old when they died. All names used in this article are fictional.

### Interviews

The first author (MAM), a PhD student and experienced intensive care nurse, carried out the interviews. Twelve interviews were conducted in the home of the bereaved, which in eleven cases was also the place of death of the partner. Two interviews were, at the request of the participants, conducted in the workplace of MAM. The interviews typically started with the bereaved spouse introducing themself, followed by the interviewer asking the bereaved to talk about the deceased partner. The participants were encouraged to talk freely, although MAM had an interview guide featuring open-ended questions to hand as guidance. The topics during the interviews were the illness of the partner and their limited lifespan, home as the preferred place of death, and how the death at home had occurred. If and how the couple had talked about the partner dying was not a specific theme; however, all the bereaved shared unsolicited experiences related to how the illness and imminent death had (not) been talked about. The duration of the interviews varied from 50 minutes to almost 3 hours. The interviews were audio-recorded and transcribed verbatim by MAM. All the participants made important contributions to the totality of the study. To substantiate the results and exemplify the different ways death was addressed, in the Results section, we present selected vivid and descriptive excerpts from the interviews.

### Data Analysis

When the first author read and re-read the transcripts to develop an overall impression of the material, her interest was drawn to the ways in which the partner’s imminent death had been spoken of/not spoken of/dealt with. The possibility and relevance of exploring this further was discussed with the co-authors. To answer the RQ, we constructed the following analytical questions (AQs): How did the bereaved describe the ways death was talked about/not talked about? How did the bereaved portray how the imminent death had been handled by themself and the dying partner?

Due to the use of reflexive thematic analysis allowing us to explore and interpret patterns across the data (Braun & Clarke, 2022), we critically engaged with the data material. Driven by the AQs, we identified relevant and often extensive excerpts from each interview, which were coded into smaller units. The labeling of the codes was either summative or interpretative (Braun & Clarke, 2022), as shown in Table 1.

**Table 1.** Example of the Summative and Interpretative Coding.

Excerpt	Codes
“It was nothing open … it was a bit of a family secret”	Summative: “nothing open,” “a family secret”
	Interpretative: “hidden and forbidden”
“We didn’t talk about it. Frank didn’t say anything about it either, but we knew. Both he and I understood …”	Summative: “didn’t talk about it,” “we knew”
	Interpretative: “shared understandings”

The codes from one transcript were used for the coding of other transcripts if they fitted the statement(s). When needed, we developed new codes. During this phase, we also focused on whether the codes were too fine-grained and narrow, potentially missing related data extracts, or too broad or general to answer the RQ (Braun & Clarke, 2022). Codes that appeared related or similar were assessed in terms of a possible merger. Guided by the AQs, the codes from all the transcripts were organized into overarching groups. Possible patterns across the material were explored, and we started to construct potential themes. Reflexive thematic analysis is not a linear process; rather, it is an iterative process that goes back and forth, allowing researchers the possibility to critically engage, re-evaluate, and re-construct the codes and themes (Braun & Clarke, 2022). After a thorough back-and-forth process, we reached a point where we had developed themes that answered the RQ, so the analytical work was ended. All the authors engaged throughout the analysis by reading the material, reflecting, and discussing it together. Through the analysis, we developed three main themes, which are presented in the Results section.

We did not use generative artificial intelligence (e.g., ChatGPT) or other technologies (e.g., NVivo) at any step during the preparation of this work.

### Ethical Considerations

The interview topic was a sensitive one for the participants, and many explained that they had not talked about what happened to its fullest extent before. Family and friends had been spared the potential burden of listening time and again, and almost no one had talked to HCPs after their partner’s death. Thus, the participants had a lot on their mind. They shared fond memories, as well as sadness, longing, doubts, and regrets, during the interview. This required the interviewer to be responsive during the interview, in addition to being attentive and willing to engage in informal discussions after the audio-recording had ended, which happened on several occasions. Before ending the interview session, the interviewer made sure that the bereaved had somebody to contact in case of distress, for example, a cancer nurse, a friend, or a family member. The participants could also contact the interviewer later if they wanted, which happened in one case.

This study was approved by the Regional Committee for Medical and Health Research Ethics of Eastern Norway (Project No. 95689) and the Norwegian Data Inspectorate for Research (Project No. 432421), and conducted in accordance with the Declaration of Helsinki (WHO, 2022). All the participants had received written information about the study in advance, and before the interviews started, the participants all signed an individual informed consent form. The audio-recordings and transcripts were stored at the University of Oslo on a secure research platform that complied with current privacy regulations. Only the authors had access to the dataset.

## Results

The bereaved described a long and loyal relationship with their deceased partner, one based on a commitment and desire to stay together in sickness and in health, until death parted them. However, they emphasized that they had also experienced disagreements and ups and downs throughout life. When the bereaved and their partner found themselves in a situation of illness and imminent death, they had relied on knowing each other well but also experienced new aspects of themself and/or their partner during that time. This had manifested as, for example, greater closeness than before or less companionship due to a non-shared understanding of the situation.

This section is structured according to the following main themes: “Idea(l)s and realities of communication,” “Different kinds of talks,” and “Unspoken understandings and showing without talking.” The first theme provides a backdrop to understanding what the bereaved expected or hoped for at the time and a background to their retrospective reflections on how death was addressed. The second theme demonstrates that discussions about death happened in many ways, from direct talk to evasiveness. The third theme illuminates how death was acknowledged and addressed, although not verbally articulated.

### Idea(l)s and Realities of Communication

The participants’ reflections on how death should have been talked about often stemmed from how dying and end-of-life conversations had been portrayed in television programs or films. Paul, who had been married to Ruth for many years, said: “I don’t know what I was thinking. What kinds of thoughts I had… I remember there was a movie I saw. It was about cancer. They were lying in bed talking deeply. That was the ideal.” His idea of connection and conversation conflicted with what had happened between him and Ruth during her final days. He continued:

I remember the last time I stroked Ruth and she kind of [Paul waves his hand dismissively] “Don’t touch!” It was just discomfort. It was painful. If I had been prepared for such a situation, it might have been easier. Suddenly, it was beyond the point where we could talk the way we used to.

Hence, the idea(l) of the ill partner peacefully fading away in a controlled manner after the couple had talked things through did not always correspond to what actually happened at the end. Furthermore, the expectation of certain ways of handling the imminent death was not always shared by the couple. Monica and Neil, who lived with their teenage children, had always worked well as a team, as Monica explained. Yet, when Neil became seriously ill, it proved challenging for them to collaborate. In an attempt to broach what lay ahead, Monica tried something she had seen in a movie:

I would have wanted him to … because I mentioned to Neil … We saw a movie after he became ill, a movie about a mother who wrote letters to her kids before she died, and I said, “What if you write something to the kids? A letter they can have when they are older. About life and stuff, as you won’t be able to have that talk.” But all things like that, it was … he didn’t want to talk about it.

Although all the bereaved had thoroughly considered how to talk with their partners while they were alive, what had been said (or not) at the time was still a concern. What the bereaved had thought to be a good or correct thing to do had been carefully balanced with what the partner had wanted. Oliva, who had lost her husband Walter, related the following:

I’ve thought a lot about whether we should have talked more about him dying and what he thought about it. Should I have asked more? Should we have talked more? But I don’t think so, because as long as Walter had hope, it would have been wrong of me to say “We have to talk about how it really is.” I think that would have been a mistake, but then again, I wish I had known more about what he was thinking.

As shown, the opportunities to engage in what were considered correct interactions and good conversations at the end of life varied. The retrospective reflections of the bereaved point to how expectations about end-of-life communication could possibly have added to concerns during an already demanding situation. In retrospect, the bereaved had either made peace with how the end-of-life conversations turned out or evaluated themself from the perspective of hindsight to have perhaps been too naive or too evasive.

### Different Kinds of Talks

When the imminent death had been talked about, the ways of doing so had varied. In a few cases, death had been addressed directly between the couple using words such as “die” and “dying,” although this had only happened occasionally. After initially being reluctant to talk about his illness and imminent death, Tom had become comfortable with talking openly about such things. As his wife Kate explained, “There were many who said, ‘I don’t see that he’s ill’, so no one believed him when he said ‘I’m going to die soon’, because he often told people ‘I will die soon.’” Kate related how Tom had normally avoided talking about difficult things but how she had insisted he do so when he became ill. This had been for his sake but also for her own. “It was too much for me to keep to myself,” she said, “I needed a lot of comfort.” According to Kate, Tom had eventually become “good at talking,” something she was grateful for.

During the illness trajectory, death had mostly been spoken of in meetings with HCPs held in hospitals. However, when the partner’s condition deteriorated, the encounters with HCPs took place in the couple’s home. Paul and Ruth had lived in a nice house in a great location for many years. Paul explained how the meetings with the HCPs in their home had become a somewhat surreal experience.

When the palliative care team came, Ruth was sitting on the sofa. I served coffee and we talked about how we lived in a beautiful place, in a fine house, you know, a nice talk. Then Ruth asked, “How much time do I have left? Months? Weeks?” And the doctor replied, “I think it’s between one and two weeks. Maybe a week.” Ruth was shocked. “A week! Here I’m sitting on the sofa and I’m going to die in a week. That can’t be right!” And then it was less than a week until she died. It was an absurd situation and I think that all the horrible thoughts, all the longing, the physical pain of dying, I think that was greatly reinforced by dying at home.

As described by Paul, conversations about dying (at home) could be demanding, but it had never been considered by Paul that Ruth should have died elsewhere. “We were just relieved that it was possible,” Paul said, “That she didn’t have to go to the hospital. That was never an option for us.” Yet the mix of the familiar (home) and the unknown (death) had been experienced as challenging.

For Monica, it had been important to address Neil’s inevitable death when he spent his end-of-life time at home, although it was only in the final hours of his life that she put her needs before his and directly addressed what was about to happen:

On the day Neil died, he could only whisper. It was hard to understand what he said. He looked at the razor and I asked if he wanted me to shave him and he nodded. I said, “Do you know that you are going to die soon? You will die soon” and he nodded.

This was, Monica explained, the closest Neil came to admitting that he was dying. It was only at the moment when death was actually near that it was possible to talk about it. Up until that point, they had been out of step due to their disparities in acknowledging the situation.

As demonstrated, some couples had used direct language to talk about the imminent death being part of their present life, how much time was left, and how death happened in the here and now. Doing so, however, had not always been straightforward. By contrast, the imminent death had in some cases been talked about between the lines. Una and Vincent had created a home they loved over the years. When Vincent became seriously ill, their relation to their esthetic home had been a way to talk about Vincent’s forthcoming death at home. Una told:

The cancer nurse visited us one winter’s day. I remember it was so lovely. We had lit a fire and I had baked. We spoke confidentiality. Vincent became very fond of her. In that conversation, it came up that we wanted … if it was me or him first, we wanted to die at home, because we thought we had such a cozy home. We didn’t plan or anything because Vincent didn’t talk much about death. He was very focused on living until his last breath.

By de-personalizing the impending death, talking about “we” instead of the dying partner, death could be kept at a distance and not allowed to become too close and personal.

Among the other ways to address death (at home) indirectly was talking about where the partner should not die. Frank, the husband of Emma, had worried that his care needs would place too much strain on her and that his unspoken hope of dying at home would not be possible. Emma recalled the situation well.

He never said anything about it really, but we had a terrible episode one night with a lot of washing and changing of clothes and bedlinen. He found it absolutely terrible! He said, “Emma, this isn’t going to work anymore. I’ll probably have to go to a nursing home,” and I shouted, and I can be quite harsh you know, “YOU’RE NOT GOING TO A NURSING HOME, FRANK!” And after that he was … he wasn’t worried about moving. I reassured him that he wasn’t going anywhere.

When Emma was asked by the interviewer if that was the closest they had come to talking about Frank’s preference for dying at home, she laughed a little and said, “Yes, I actually think so.” Despite being a vague approach to the partner’s imminent death, it appeared that indirect talks fostered a shared understanding of what was happening. However, for some bereaved, the partner’s reluctance to speak directly had been difficult to come to terms with. Monica, for example, had received a list of dos and don’ts for the future from Neil.

Neil told me, admonished me, “Don’t have a big funeral. Don’t remarry. Don’t waste the children’s money.” So, in a way, he had thought about death, but we never talked about it. There was a lot of indirect talk about death.

It sometimes happened that the illness and/or imminent death had been actively rejected. On some occasions, the partner’s refusal to talk about death was understood as a habitual way of approaching illness and death in general. Jakob had been a sociable and jovial man, as his wife Irma explained. He had liked to make a joke, and his dismissal of so-called serious talk was understood to be part of his personality. For him, the circumstance of him dying and how his death should happen at home was self-evident, meaning that there was nothing to talk about. As Irma noted:

He said, “I understand what’s happening,” but he couldn’t talk about it. “I’m not stupid, I feel it in my body,” he told us. I don’t think he knew any other way to do this. He hated hospitals, and he hated nursing homes. He didn’t want to talk about illness at all. He had never liked to deal with illness and hospitals and stuff like that. “There must be something else to talk about,” he said. He couldn’t bear listen to that kind of talk. “Let’s talk about something else,” he always said.

Some partners’ had chosen to only talk to others than the spouse about their imminent death. Gabriel, who had been married to Helen, explained how he had overheard a conversation Helen had had with a friend, which meant he partly knew how she felt about dying.

Once, I completely broke down and cried, and Helen got upset. She shouted: “Stop! Stop it!” so I did. I don’t know if I did the right thing, but I think she didn’t want me to break down or feel that way. We never talked about emotions. I asked her once if we should look at pictures, look at all the memories throughout life. “No,” she said, “you can do that when I’m gone.” She didn’t want to approach death and talk about it, at least that’s how I understood it, so we didn’t talk about it much. I only remember things she talked about when she had visitors. She said, “I’m not afraid of dying, but I think it’s sad not to be around anymore.”

As shown, in some contexts, death was allowed into certain conversations but not into others. Perhaps it was when the most was at stake that death was held in the background, at a distance, kept away from near ones.

When, following the directive of the partner, death had not only been avoided but also banned from being discussed, it had been challenging because the bereaved had had to balance the wish of the dying partner, caring for the rest of the family, and their own needs. Earlier experiences of the bereaved concerning death and mourning also affected what they considered to be ideal communication. As Monica recalled:

I asked for a family appointment at the hospital. There was a doctor and a nurse, and the nurse turned to the children and said, “What’s it like to know that dad is seriously ill?” But Neil said, “I’m not seriously ill,” and that set the tone for the children. “This isn’t something to ask about,” he said. He didn’t want that. He wasn’t ill. Didn’t want to focus on that. I thought, “He’s seriously ill, so he’s wrong about that.”

Discussion about the situation had been closed down, and at the time, it was not possible to approach the fact that Neil was dying. When reflecting on the situation in retrospect, Monica offered the following:

We just had to respect that he didn’t want to talk, but I think that was a way of letting the children down. And me. There was no climate to talk about it at home. It was nothing open, it was a bit of a family secret. We were isolated. It’s sad. It would have been nice if we could have talked about it, but I don’t think he was able to. It was difficult and still is very difficult. We have the youngsters who must deal with this and live on … it’s difficult. If I were to do it again, I don’t think I’d have been as respectful to be honest, because it caused so many problems afterwards. I would probably have pushed a little harder.

With young children at home, the expectation of talking (more) appeared even more important, both to understand what was happening in the here and now and also to facilitate future processes concerning death and loss.

We have shown that discussions about the partner’s death happened in various ways and that illness and death could also be banned as topics of conversation. In the next subsection, we will examine how the (imminent) death was communicated without talking.

### Unspoken Understandings and Showing Without Talking

Some bereaved talked about how they and their partner had shared a common understanding about death, a kind of shared knowing-without-saying, a situation where spoken words had been redundant. As Emma explained:

There was nothing said between us like “Now it’s coming to an end.” We didn’t talk about it. Frank didn’t say anything about it either, but we knew. Both he and I understood … It was so obvious that there wasn’t much to talk about. We knew.

The “knowing” came from seeing and understanding the partner’s symptoms, for example, reduced appetite, decreased mobility, and drifting in and out of consciousness, as well as from the bodily experience of the partner. In addition, the couples had shared their life together for a long time, and their strong sense of unity also played a part in “just knowing.” Pia and Simon had, according to Pia, only talked about Simon dying once:

We didn’t really talk about it. We knew each other so well and agreed that we just wanted to be at home. I think it’s almost a coincidence that he died at home because we didn’t talk about it. We didn’t talk about death, except for that one time at the hospital.

As demonstrated, there was not always a need for words. The couple knew what was happening, and they were connected in having a shared understanding about the imminent death without talking about it.

Some partners had shown an understanding and awareness of their imminent death through doings rather than by talking. For instance, by making sure there was enough firewood for the winter season or by picking out which clothes to be buried in. The things that had been done by the partners at the end-of-life stage to make it easier on those who would be left behind was understood by the bereaved as a great act of care. Monica related with both gratefulness and sadness how Neil had arranged things for the family:

Neil cleaned up the garage. Sold the trailer and the chainsaw and things like that. A sort of cleaning up in life to make things easier, right? He did so much to make it easier for us, so he had, in a sense, a very high awareness that he wouldn’t be around, but he couldn’t talk about it. He was left so alone with everything when we could have been together. I guess he wanted to spare us, but he only made it worse.

The loneliness and disconnection at the end of life were, in retrospect, perceived as unnecessary and meaningless, as a burden to bear. Instead of giving the family an opportunity to be close at the end of life, something the home environment could have facilitated, the non-addressed yet imminent presence of death occasionally caused distance.

Jacob was one of those who had decided on the clothes he wished to be buried in—the uniform of his sports club. He was preparing for death, parting, and loss, something that re-shaped his interactions with his wife. As Irma explained:

After Jacob became ill, he became close in a different way. He used to say that he loved me, but there was a difference in how he treated me. When we had dinner, he sat beside me at the table and stroked my hand. He didn’t normally do that.

As shown above, there were different ways of addressing death and dying. The way death had been communicated was related to the individuals involved, the timing, and the setting—specific situations where acknowledging the limited time remaining may have been less daunting. Couples appeared to find common ground more readily through non-verbal (inter)actions rather than open and direct conversations about death. What had caused challenges during the time before death, and also after the death of the partner, was when the way death had been addressed had been different to what the bereaved had expected or needed, which had triggered a sense of solitude and distance.

## Discussion

This study explored how the bereaved reflected on and described the ways death had been addressed when their partner spent their end-of-life period at home and then died there. We found that circulating and collective narratives about how death should be talked about had impacted the couples’ interactions at the time and were still something that the bereaved pondered over—should we have talked more? Death had been addressed both verbally and non-verbally, although the verbal communication had not been equal to open conversations. A more common way of talking about death was indirectly or in an avoidant way. The non-verbal approach came to light through actions and was understood by the bereaved as their partner’s way of addressing and acknowledging their imminent death without talking about it. While some bereaved had hoped for more or other forms of conversations, all believed that their partner had approached the existential dimension of death—that is, the inevitable and definitive separation from loved ones and life itself—as best they could, considering their personality and the overall situation. The practical aspects related to death, for example, what to wear when being buried or where to place the hospital bed at home, were easier to address than the painful reality of losing each other for ever.

Sharing personal experiences of illness and death has become increasingly normalized in our society and is often depicted as both a moral and existential good (Thorbjørnsrud & Ytreberg, 2020; Thoresen & Rugseth, 2022). Due to modern communication technology and the increased social acceptance of and interest in (personal) illness narratives, there has also been a growing culture of sharing since the 1990s related to living with a cancer diagnosis and/or being a cancer survivor (Kvaale et al., 2024). Both true and fictive stories of illness and death surround us and can be used for consolation and motivation. Some of the bereaved who participated in the present study explained that they had been influenced by portrayals of end-of-life interactions in the media, where the ethos of openness (Thorbjørnsrud & Ytreberg, 2020) was highlighted. In the fictive scenarios, the ill and dying person was often depicted as a responsible individual when their life was nearing the end, someone who was willing to reminisce about shared experiences, openly acknowledge the situation in the here and now, and facilitate the subsequent life of those who would shortly be left behind. Trying to transfer and apply fiction to one’s own life might be understood as a naive approach, but it could also be interpreted as a need for guidance and for something to navigate by in a challenging situation. Caring for a dying family member has been described as involving entering an unknown terrain relationally, emotionally, and practically (Aurén-Møkleby et al., 2023), a situation where a lack of good communication between the patient, their next of kin, and the HCPs can cause anxiety, insecurity, and reduced preparedness for what to expect at the end of life (Barlund et al., 2021). This accords with the reflections of some of the bereaved in the present study, who experienced a loss of relational connection. Emotionally close individuals may assume they understand each other’s thoughts on difficult matters, such as severe illness and dying. When these assumptions don’t hold, they not only face an uncertain and frightening situation but also lose their familiar way of being “us.”

Open awareness—that is, “giving the patient an opportunity to close his life in accordance with his own ideas about proper dying” (Glaser & Strauss, 1965, p. 103)—has been highlighted as a way of arranging for a good death. The idea of a proper or good death has been modified as further knowledge has added to the understanding of dying, and in a recently published article, Pollock et al. (2024) convincingly challenged the construction of the good death “as a way of disciplining and constraining patient and public perspectives through the promotion of normative ‘choices’ about ‘dying well’” (Pollock et al., 2024, p. 10). Based on the findings of our study, we are inclined to assert that the idea(l) some of the bereaved espoused about “the good talk” was associated with the thought of a good death for their partner, something that possibly added to their concerns during an already distressing time. Most couples did not have an open dialogue about the imminent death and yet the partner died at home—“almost a coincidence,” as some of the bereaved noted. In the present study, the dying partners were more inclined to choose other ways to address their illness and impending death than through conversations. This differs from the findings of a systematic review on cancer-related communication within couples, where patients tended to communicate more openly than their spouses, although there was inconsistency regarding both role and gender concerning the degree of communication (Hasson-Ohayon et al., 2022).

Regardless of how death was addressed, based on the experiences of the bereaved in our study, we argue that all the partners had an awareness that they were seriously ill and about to die. When this was not expressed in words, it came to light through actions, such as cleaning out the garage. These findings point to how the partners had an opportunity to choose the degree to which illness and death were “invited” into their life, which resembles how Arantzamendi et al. (2020) describe how awareness of dying can be put in the background or foreground. According to them, “How the participants positioned their Awareness of Dying determined how it influenced the process of living well with advanced cancer” (Arantzamendi et al., 2020, p. 1150). Living well for a limited time was also a core matter in the present study, and the future-related preparations by the partner could be understood as a way of doing just that. In other words, the dying partner took charge and responsibility, and the bereaved experienced what was done as acts of care and love, sparing them from extra work in their subsequent life.

Talking about thoughts and possible fears of dying can be helpful when all involved are willing to do so (Walter, 2017). The bereaved in this study were attentive to whether their dying partner felt comfortable engaging in such conversations or not. Despite most bereaved believing that they had done the right thing by not pushing ahead with conversations about death, there were still doubts about whether it had been right or not to hold back. This was particularly apparent when there had been children living at home, where a more open approach might have made it easier for the whole family to share and handle the death of the other parent. The benefits of generally being more open regarding illness and death are unclear, and Goldsmith and Miller (2014) address this when conceptualizing how couples talk about cancer, underlining “If we wish to advise couples to talk, we need to know what they should talk about and how” (Goldsmith & Miller, 2014, p. 51). In the era of openness about death, the “what,” “how,” and “to whom” must be considered and adjusted based on the people involved, time, and context. The sociologist Tony Walter (2017) discusses how talking about death online or in a death café with strangers does not automatically facilitate openness within one’s own family. Confirming a desire to talk about death, which can be done in such fora, could possibly distance people from their own family, where death is not a topic of conversation. “We simply do not know,” Walter (2017, p. 25) concludes.

Today, more people with cancer receive end-of-life care at home, and the help and support they receive from family members when navigating the dying process and death trajectory are vital (Kaasa et al., 2018; Sallnow et al., 2022). For some, the concept of death literacy, which can be described as “a set of knowledge and skills that make it possible to gain access to, understand, and act upon end-of-life and care options” (Noonan et al., 2016, p. 2), can be helpful when it comes to meeting the needs of dying people and their next of kin. However, while we agree that concepts such as death over dinner, where family and friends can face “the frightening—talking about death—and transforming it into the familiar—a conversation over dinner” (Lambert South & Elton, 2017), similar to what Barbie did, can prove positive, we consider a theoretical and philosophical conversation about death in such a context to be quite different from emotional and existential discussions at home when disruption and parting are the reality and lived experience (Keeley & Generous, 2017). In a prior study about how couples talk about cancer, it was found that the most difficult topic for couples to discuss was death, followed by sex (Goldsmith & Miller, 2014). The findings of the present study add to this—death is difficult to talk about. After all, “the ability to learn to live with death is still – and perhaps will remain – one of the most daunting and difficult tasks with which humanity has to deal” (Jacobsen, 2016, p. 17).

### Strengths and Limitations

We consider it a strength that many bereaved wanted to share their experiences and boldly did so. Their willingness to talk about what they had experienced also pointed to an unmet need on the part of the bereaved after their partner’s death. What had been said/not said did not rest in peace for all; rather, it was still a concern, something that has clear implications for practice. This study relied solely on the recollections and accounts of the bereaved, thereby conveying their perspective on how death was addressed when their partner died at home. What their now deceased partner did or did not do is hence framed and interpreted from the perspective of the bereaved. Those who are now dead would possibly have related things differently, emphasizing other matters. Nonetheless, what is brought to light in the present study can aid in comprehending how couples handle living together when one is close to death and extend the understanding of how awareness of dying can be expressed in various ways, something that has little to do with a “good way” whether it comes to addressing death or to dying itself. Although this study focused on cancer and the context of home death, it illuminates the varied ways in which couples might interact when faced with illness or other distressing life events. Therefore, we believe that the relational dynamics highlighted by our findings are applicable and relevant to other contexts.

### Final Remarks

Idea(l)s about open dialogue may set unrealistic expectations concerning dying people and their partners when it comes to talking and interacting in a certain way at the end of life. Awareness of dying can be expressed in various ways, where words such as “death” and “dying” are sometimes redundant or unspeakable. Thus, the way of addressing death and dying cannot be routinized; instead, it must be figured out by those involved, based on their relationship, history, and understanding.

## Note

1. Our translation.

## References

[bibr1-10497323251328294] Ågren A. Krevers B. Cedersund E. Nedlund A.-C. (2023). Policy narratives on palliative care in Sweden 1974–2018. Health Care Analysis, 31(2), 99–113. 10.1007/s10728-022-00449-1 36650304 PMC10126030

[bibr2-10497323251328294] Arantzamendi M. García-Rueda N. Carvajal A. Robinson C. A. (2020). People with advanced cancer: The process of living well with awareness of dying. Qualitative Health Research, 30(8), 1143–1155. 10.1177/1049732318816298 30539681 PMC7307002

[bibr3-10497323251328294] Ariès P. (1976a). Forbidden death. In Ranum P. M. (Trans.), Western attitudes toward death from the middle ages to the present (pp. 85–107). Marion Boyars Publishers.

[bibr4-10497323251328294] Ariès P. (1976b). Western attitudes toward death: From the middle ages to the present. Marion Boyars.

[bibr5-10497323251328294] Aurén-Møkleby M. Aasbø G. Fredheim O. M. S. Mengshoel A. M. Solbrække K. N. Thoresen L. (2024). “It turned out right for both of us”: A qualitative study about a preference for home death and actual place of death. Death Studies. Advance online publication. 10.1080/07481187.2024.2369847 38916193

[bibr6-10497323251328294] Aurén-Møkleby M. Thoresen L. Mengshoel A. M. Solbrække K. N. Aasbø G. (2023). ‘It’s not just about me’: A qualitative study of couples’ narratives about home death when one of the partners is dying of cancer. Palliative Care and Social Practice, 17. 10.1177/26323524231189517 PMC10399270 37545874

[bibr7-10497323251328294] Barlund A. S. André B. Sand K. Brenne A.-T. (2021). A qualitative study of bereaved family caregivers: Feeling of security, facilitators and barriers for rural home care and death for persons with advanced cancer. BMC Palliative Care, 20(1), Article 7. 10.1186/s12904-020-00705-y 33419428 PMC7796575

[bibr8-10497323251328294] Borgstrom E. Walter T. (2015). Choice and compassion at the end of life: A critical analysis of recent English policy discourse. Social Science & Medicine, 136–137, 99–105. 10.1016/j.socscimed.2015.05.013 25989003

[bibr9-10497323251328294] Braun V. Clarke V. (2013). Successful qualitative research - A practical guide for beginners. Sage.

[bibr10-10497323251328294] Braun V. Clarke V. (2022). Thematic analysis: A practical guide. Sage.

[bibr11-10497323251328294] Burr V. (2015). Social constructionism (3rd ed.). Routledge.

[bibr12-10497323251328294] Clark D. Seymour J. (1999). Reflections on palliative care. The Open University Press.

[bibr13-10497323251328294] Driller B. Talseth-Palmer B. Hole T. Strømskag K. E. Brenne A.-T. (2022). Cancer patients spend more time at home and more often die at home with advance care planning conversations in primary health care: A retrospective observational cohort study. BMC Palliative Care, 21(1), Article 61. 10.1186/s12904-022-00952-1 35501797 PMC9063101

[bibr14-10497323251328294] Gardner D. S. (2008). Cancer in a dyadic context: Older couples’ negotiation of ambiguity and search for meaning at the end of life. Journal of Social Work in End-of-Life & Palliative Care, 4(2), 135–159. 10.1080/15524250802353959 19042897

[bibr15-10497323251328294] Gerwig G. (2023). Barbie. Warner Bros.

[bibr16-10497323251328294] Glaser B. G. Strauss A. L. (1965). Awareness of dying. Routledge Taylor & Francis Group.

[bibr17-10497323251328294] Goldsmith D. J. Miller G. A. (2014). Conceptualizing how couples talk about cancer. Health Communication, 29(1), 51–63. 10.1080/10410236.2012.717215 23442190

[bibr18-10497323251328294] Haaksman M. Ham L. Brom L. Baars A. van Basten J.-P. van den Borne B. E. Hendriks M. P. de Jong W. K. van Laarhoven H. W. van Lindert A. S. Mandigers C. M. P. W. van der Padt-Pruijsten A. Smilde T. J. van Zuylen L. C. van Vliet L. M. Raijmakers N. J. H. (2024). Open communication between patients and relatives about illness & death in advanced cancer—Results of the eQuiPe Study. Supportive Care in Cancer, 32(4), Article 214. 10.1007/s00520-024-08379-5 38446248 PMC10917842

[bibr19-10497323251328294] Häger Tibell L. Årestedt K. Holm M. Wallin V. Steineck G. Hudson P. Kreicbergs U. Alvariza A. (2024). Preparedness for caregiving and preparedness for death: Associations and modifiable thereafter factors among family caregivers of patients with advanced cancer in specialized home care. Death Studies, 48(4), 407–416. 10.1080/07481187.2023.2231388 37441803

[bibr20-10497323251328294] Hasson-Ohayon I. Goldzweig G. Braun M. Hagedoorn M. (2022). Beyond “being open about it”: A systematic review on cancer related communication within couples. Clinical Psychology Review, 96, Article 102176. 10.1016/j.cpr.2022.102176 35700574

[bibr21-10497323251328294] HospiceUK . (2024). Dying matters. https://www.hospiceuk.org/our-campaigns/dying-matters

[bibr100-10497323251328294] Järvinen M. (2017). Symbolsk interaktionisme som analysestrategi [Symbolic interactionism as an analysis strategy]. In Järvinen M. Mik-Meyer N. (Eds.), Kvalitativ analyse - syv traditioner [Qualitative analysis - Seven traditions] (1st ed., pp. 27–55). Hans Reitzels forlag.

[bibr22-10497323251328294] Jacobsen M. H. (2016). “Spectacular death”—Proposing a new fifth phase to Philippe Ariès’s admirable history of death. Humanities, 5(2), Article 19. 10.3390/h5020019

[bibr23-10497323251328294] Jacobsen M. H. (2020). The age of spectacular death. Taylor & Francis Group.

[bibr24-10497323251328294] Jacobsen M. H. Dalgaard K. M. (2013). Two faces of death - ‘good’ and ‘bad’ deaths in contemporary palliative care. In Hviid Jacobsen M. (Ed.), Deconstructing death (pp. 309–329). University Press of Southern Denmark.

[bibr25-10497323251328294] Kaasa S. Loge J. H. Aapro M. Albreht T. Anderson R. Bruera E. Brunelli C. Caraceni A. Cervantes A. Currow D. C. Deliens L. Fallon M. Gómez-Batiste X. Grotmol K. S. Hannon B. Haugen D. F. Higginson I. J. Hjermstad M. J. Hui D. Lundeby T. (2018). Integration of oncology and palliative care: A Lancet Oncology Commission. The Lancet Oncology, 19(11), e588–e653. 10.1016/S1470-2045(18)30415-7 30344075

[bibr26-10497323251328294] Kastenbaum R. (1979). “Healthy dying”: A paradoxical quest continues. Journal of Social Issues, 35(1), 185–206. 10.1111/j.1540-4560.1979.tb00794.x 11662784

[bibr27-10497323251328294] Keeley M. P. (2007). ‘Turning toward death together’: The functions of messages during final conversations in close relationships. Journal of Social and Personal Relationships, 24(2), 225–253. 10.1177/0265407507075412

[bibr28-10497323251328294] Keeley M. P. (2017). Family communication at the end of life. Behavioral Sciences, 7(3), Article 45. 10.3390/bs7030045 28708107 PMC5618053

[bibr29-10497323251328294] Keeley M. P. Generous M. A. (2017). Final conversations: Overview and practical implications for patients, families, and healthcare workers. Behavioral Sciences, 7(2), Article 17. 10.3390/bs7020017 28379179 PMC5485447

[bibr30-10497323251328294] Kvaale K. Lian O. S. Bondevik H. (2024). Beyond my control’: Dealing with the existential uncertainty of cancer in online texts. Illness, Crisis & Loss, 32(2), 192–208. 10.1177/10541373221122874

[bibr31-10497323251328294] Lambert South A. Elton J. (2017). Contradictions and promise for end-of-life communication among family and friends: Death over dinner conversations. Behavioral Sciences, 7(2), Article 24. 10.3390/bs7020024 28425929 PMC5485454

[bibr32-10497323251328294] Malterud K. Siersma V. D. Guassora A. D. (2016). Sample size in qualitative interview studies: Guided by information power. Qualitative Health Research, 26(13), 1753–1760. 10.1177/1049732315617444 26613970

[bibr33-10497323251328294] Möllerberg M.-L. Sandgren A. Swahnberg K. Benzein E. (2017). Familial interaction patterns during the palliative phase of a family member living with cancer. Journal of Hospice and Palliative Nursing, 19(1), 67–74. 10.1097/NJH.0000000000000310

[bibr34-10497323251328294] Noonan K. Horsfall D. Leonard R. Rosenberg J. (2016). Developing death literacy. Progress in Palliative Care, 24(1), 31–35. 10.1080/09699260.2015.1103498

[bibr35-10497323251328294] Novack D. H. Plumer R. Smith R. L. Ochitill H. Morrow G. R. Bennett J. M. (1979). Changes in physicians’ attitudes toward telling the cancer patient. JAMA, 241(9), 897–900. 10.1001/jama.1979.03290350017012 762865

[bibr36-10497323251328294] Pollock K. Caswell G. Turner N. Wilson E. (2024). ‘Beyond the reach of palliative care’: A qualitative study of patient and public experiences and anticipation of death and dying. Qualitative Health Research, 34(14), 1428–1441. 10.1177/10497323241246705 38904368 PMC11580323

[bibr37-10497323251328294] Regjeringen . (2020). Meld. St. 24 (2019-2020). Lindrende behandling og omsorg. Vi skal alle dø en dag. Men alle andre dager skal vi leve [Palliative care. We will all die one day. But every other day we will live]. https://www.regjeringen.no/contentassets/52d05db7090c411abc7a3f4d47124119/no/pdfs/stm201920200024000dddpdfs.pdf

[bibr38-10497323251328294] Sallnow L. Smith R. Ahmedzai S. H. Bhadelia A. Chamberlain C. Cong Y. Doble B. Dullie L. Durie R. Finkelstein E. A. Guglani S. Hodson M. Husebø B. S. Kellehear A. Kitzinger C. Knaul F. M. Murray S. A. Neuberger J. O’Mahony S. Lancet Commission on the Value of Death . (2022). Report of the Lancet Commission on the value of death: Bringing death back into life. The Lancet, 399(10327), 837–884. 10.1016/S0140-6736(21)02314-X PMC8803389 35114146

[bibr39-10497323251328294] Seale C. (1998). Constructing death: The sociology of dying and bereavement. Cambridge University Press.

[bibr40-10497323251328294] Seale C. Addington-Hall J. McCarthy M. (1997). Awareness of dying: Prevalence, causes and consequences. Social Science & Medicine, 45(3), 477–484. 10.1016/S0277-9536(96)00379-6 9232741

[bibr41-10497323251328294] Taylor C. (1992). The ethics of authenticity. Harvard University Press.

[bibr42-10497323251328294] Thorbjørnsrud K. Ytreberg E. (2020). A human interest economy: The strategic value of turning ordinary people into exemplars in the news media. Journalism Studies, 21(8), 1093–1108. 10.1080/1461670X.2020.1720520

[bibr43-10497323251328294] Thoresen L. Røberg A.-S. B. (2022). The construction of the responsible patient in complex palliative care: Interpreting palliative care policies. Palliative Care and Social Practice, 16. 10.1177/26323524221118586 PMC9434665 36059854

[bibr44-10497323251328294] Thoresen L. Rugseth G. (2022). “Kommer noen til å dø?”: Om den medierte åpenheten om døden [Is someone going to die?”: On the mediated openness about death]. Tidskrift for Forskning i Sygdom og Samfund, 19(37), 43–63. 10.7146/tfss.v19i37.1

[bibr45-10497323251328294] Wallace C. L. (2015). Family communication and decision making at the end of life: A literature review. Palliative & Supportive Care, 13(3), 815–825. 10.1017/S1478951514000388 24774221

[bibr46-10497323251328294] Walter T. (1994). The revival of death. Routledge.

[bibr47-10497323251328294] Walter T. (2017). What death means now. Thinking critically about dying and grieving. Policy Press.

[bibr48-10497323251328294] WHO . (2022). WMA declaration of Helsinki- ethical principles for medical research involving human subjects. 19886379

